# Shrub canopies influence soil temperatures but not nutrient dynamics: An experimental test of tundra snow–shrub interactions

**DOI:** 10.1002/ece3.710

**Published:** 2013-09-07

**Authors:** Isla H Myers-Smith, David S Hik

**Affiliations:** Department of Biological Sciences, University of AlbertaCW405 Biological Sciences Bldg., Edmonton, Alberta, T6G 2E9

**Keywords:** Alpine, arctic, birch (*Betula)*, carbon, litter, nitrogen, permafrost, plant–soil (belowground) interactions, soil respiration, willow (*Salix*)

## Abstract

Shrubs are the largest plant life form in tundra ecosystems; therefore, any changes in the abundance of shrubs will feedback to influence biodiversity, ecosystem function, and climate. The snow–shrub hypothesis asserts that shrub canopies trap snow and insulate soils in winter, increasing the rates of nutrient cycling to create a positive feedback to shrub expansion. However, previous work has not been able to separate the abiotic from the biotic influences of shrub canopies. We conducted a 3-year factorial experiment to determine the influences of canopies on soil temperatures and nutrient cycling parameters by removing ∼0.5 m high willow (*Salix* spp.) and birch (*Betula glandulosa*) shrubs, creating artificial shrub canopies and comparing these manipulations to nearby open tundra and shrub patches. Soil temperatures were 4–5°C warmer in January, and 2°C cooler in July under shrub cover. Natural shrub plots had 14–33 cm more snow in January than adjacent open tundra plots. Snow cover and soil temperatures were similar in the manipulated plots when compared with the respective unmanipulated treatments, indicating that shrub canopy cover was a dominant factor influencing the soil thermal regime. Conversely, we found no strong evidence of increased soil decomposition, CO_2_ fluxes, or nitrate or ammonia adsorbtion under artificial shrub canopy treatments when compared with unmanipulated open tundra. Our results suggest that the abiotic influences of shrub canopy cover alone on nutrient dynamics are weaker than previously asserted.

## Introduction

Foundation species that form the dominant architecture and structure of an ecosystem can also act as ecosystem engineers influencing ecosystem functions via multiple causal pathways (Ellison et al. 2005; Angelini et al. 2011). For example, forest tree species provide shade, deposit litter, and alter microclimates and thus, influence decomposition, nutrient fluxes, carbon sequestration and energy flow (Ellison et al. 2005; Angelini et al. 2011).

A change in the dominance of canopy-forming species can alter ecosystem functioning. Shrub, bush, or scrub canopies are increasing in a variety of ecosystems worldwide including temperate grasslands (Van Auken 2000, 2009; Knapp et al. 2008), African savannas (Archer et al. 1995; Roques et al. 2001) and Arctic and alpine tundra ecosystems (Myers-Smith et al. 2011a; Naito and Cairns 2011; Brandt et al. 2013). A significant increase in shrub cover in these ecosystems has the potential to dramatically alter the microclimate, nutrient cycling, and species composition (Knapp et al. 2008; Myers-Smith et al. 2011a). However, manipulative experiments are rarely conducted on tall shrub species and thus we have a limited understanding of how the abiotic properties of canopies act to control ecosystem functions and biodiversity.

Tundra ecosystems are predicted to undergo a variety of rapid ecological changes with warming (Post et al. 2009) including permafrost thaw (Schuur et al. 2008) and more frequent tundra fires (Mack et al. 2011); however, perhaps the most prominent ongoing terrestrial change is the widespread increases in the cover of shrub species (Myers-Smith et al. 2011a). Repeat photography documents an increase in *Alnus viridis* in northern Alaska (Sturm et al. 2001b; Tape et al. 2006), and a variety of willow species in the western Canadian Arctic (Lantz et al. 2009, 2010; Mackay and Burn 2011; Myers-Smith et al. 2011b) and Arctic Russia (Forbes et al. 2010; Macias-Fauria et al. 2012). Population age distributions indicate recent up-slope advances of *Juniperus nana* in sub-Arctic Sweden (Hallinger et al. 2010) and willow species the Yukon Territory (Danby and Hik 2007b; Myers-Smith 2011). Increases in tall shrub species have been observed in long-term monitoring plots at many sites around the circumpolar Arctic (Elmendorf et al. 2012); however, shrub cover is not increasing at all Arctic sites (Daniëls et al. 2011; Myers-Smith et al. 2011a; Elmendorf et al. 2012; Tape et al. 2012). Widespread increases in woody shrub species around the circumpolar north will alter abiotic and biotic ecosystem processes, and could generate positive feedbacks to future shrub expansion and further climate warming (Sturm et al. 2001a, 2005). Tundra ecosystems are, therefore, an ideal place to explore the interactions between ecosystem structure and function, and to determine how the increase in tall shrub species can create feedbacks to alter ecosystem processes and future vegetation change.

Shrub canopies play major roles in the functioning of many ecosystems by influencing light penetration, soil moisture, and fire frequency in the surrounding environment (Knapp et al. 2008). In tundra ecosystems, shrub canopies also alter snow accumulation, distribution, physical characteristics, melt, and permafrost thaw (Sturm et al. 2001a; Pomeroy et al. 2006; Blok et al. 2010; Marsh et al. 2010). In winter, subnivian temperatures under shrub canopies that trap snow can be as much as 30°C warmer than air temperature (Sturm et al. 2005), and these warmer temperatures can potentially enhance winter nitrogen cycling and lead to the release of larger pulses of nitrogen in spring (Weintraub and Schimel 2003, 2005; Buckeridge and Grogan 2010). During spring, shrub stems extend above the snowpack and can alter albedo and accelerate local snow melt (Sturm et al. 2001a; Sturm 2005; Pomeroy et al. 2006; Loranty et al. 2011). In contrast, in summer, shading under shrub canopies reduces soil temperatures (Marsh et al. 2010) and active layer depth (Blok et al. 2010). The abiotic influences of shrub canopies on soil temperatures could therefore alter biotic ecosystem functions such as decomposition, nutrient cycling, and plant growth.

Snow–shrub interactions have been hypothesized to create positive feedbacks to shrub growth and expansion by increasing nutrient availability in soils under shrub canopies (Sturm et al. 2001a, 2005; Grogan and Jonasson 2006). By trapping snow, shrub canopies are thought to accelerate nutrient cycling, thereby enhancing nutrient availability (Weintraub and Schimel 2005). Fertilization experiments show that vascular plant productivity is nutrient limited in tundra ecosystems (Shaver and Chapin 1980; Mack et al. 2004), and both nitrogen fertilization experiments and warming experiments in tundra have resulted in increased biomass of shrub species (Dormann and Woodin 2002). In addition, larger inputs of higher quality leaf litter under tall birch canopies have also been shown to promote rapid soil nitrogen cycling in birch tundra (Buckeridge et al. 2010). Conversely, shrub increases in tundra ecosystems have been predicted to reduce soil decomposition rates (Cornelissen et al. 2007) because deciduous shrub litter has been reported to be more recalcitrant than herbaceous litter (Hobbie 1996; Cornelissen et al. 2007; Baptist et al. 2010), with woody plants potentially allocating more carbon to recalcitrant forms such as lignin, and producing more polyphenols and tannins which can retard decomposition (De Deyn et al. 2008). These contrasting results suggest that if the biomass of shrub species increases in tundra ecosystems, decomposition, nutrient cycling, and nitrogen availability could create either a positive or negative feedback to shrub growth.

Previous explorations of snow cover and nutrient cycling have involved snow manipulation experiments or snow depth gradient studies. The studies have demonstrated that deeper snow depth can increase litter decomposition (Baptist et al. 2010), nutrient cycling and spring nitrogen pulses (Schimel et al. 2004; Nobrega and Grogan 2007; Buckeridge and Grogan 2010), summer nitrogen mineralization rates (DeMarco et al. 2011), and can alter soil microbial communities (Chu et al. 2011). However, snow fence studies usually create deeper snow depth manipulations than the increases in snow cover likely to occur with shrub expansion (Wipf and Rixen 2010).

Great uncertainty still remains about the temperature sensitivity of soil carbon decomposition and potential feedbacks to climate warming (Davidson and Janssens 2006), and in particular the role of vegetation in regulating soil temperatures and altering biogeochemical cycles. Tundra soils store large quantities of carbon, in the range of 1400–1850 Pg C in the northern cryosphere region (McGuire et al. 2010), and are important components of global carbon budgets (McGuire et al. 2009). These carbon stores are currently protected by cold soil temperatures and permafrost, which slow microbial decomposition and the release of carbon into the atmosphere. However, with climate warming, permafrost thaw and changes in vegetation cover, this stored carbon could become vulnerable to decomposition (Mack et al. 2004; Schuur et al. 2009). Therefore, a better understanding of plant–soil–climate feedbacks, with particular reference to changing shrub cover, will improve models and predictions of the impacts of future climate on tundra ecosystem function (Chapin et al. 2009; Euskirchen et al. 2009).

Though the “snow–shrub hypothesis” (Sturm et al. 2001a, 2005) is currently widely accepted, no experimental tests exist using artificial canopies. In this study, we established a fully factorial manipulative experiment by removing natural shrub canopies and creating artificial canopies over previously shrub-free tundra. Using this approach, we tested the influence of shrub canopies in isolation from soil conditions or plant communities, which differ between shrub and shrub-free tundra. Artificial canopies have been used in desert ecosystems to test the influence of shading and water availability on understory species (Holzapfel and Mahall 1999), but have yet to be employed in tundra ecosystems. With this manipulative experiment we explore the following research questions: (1) Do shrub canopies insulate soils by trapping snow in winter and shading soils in summer? (2) Does shrub canopy cover explain variation in nutrient parameters? and, (3) which abiotic or biotic factors associated with shrub canopy cover best explain variation in nutrient dynamics?

## Methods

### Study site

**Figure 1:**
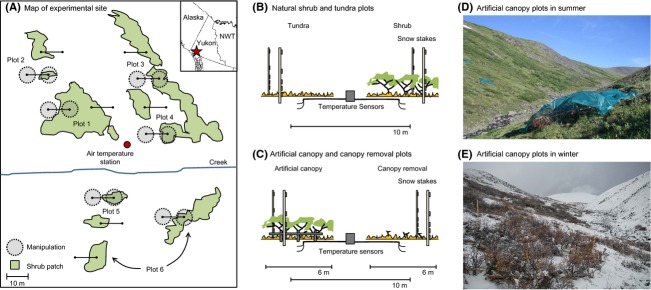
The location of experimental plots (A), the design of the canopy manipulation (B and C), and the artificial canopy treatments in summer (D: plot 5, B) and winter (E: plot 4, A). The inset indicates the general location of the study site in the Yukon Territory (61.22°N, 138.28°W, at 1450 m a.s.l.). The dashed gray circles represent the manipulated artificial canopy and canopy removal treatments and the green polygons are shrub patches.

We conducted our experimental manipulation in alpine tundra with a landscape mosaic of approximately 50% cover of shrub patches with canopy heights of 30–100 cm. Our experimental site (61.22°N, 138.28°W, at 1450 m a.s.l.) was located on either side of a stream that bisected a valley with east- (18° slope) and west- (23° slope) facing slopes in the Ruby Range Mountains of the Kluane Region, southwest Yukon Territory, Canada (Fig. 1). This region has a mean annual temperature of −3.8°C, with an annual average rainfall of 192 mm and an average annual snow fall of 106 cm (Environment Canada Burwash Weather Station). The dominant tall shrubs at the site were the willow species *Salix pulchra* Cham., *Salix glauca* L. Hook., and *Salix richardsonii* Hook. Common understory species include *Salix reticulata* L., *Dryas octopetala* L., *Polygonum bistorta* L. ssp. *plumosum* (Small) Hultén, *Festuca* spp., *Carex* spp. and moss and lichen species (Cody 2000). Plant species composition and biomass varied between shrub and open tundra plots (Fig. 5). Soils were 5–50 cm deep organic cryosols (Canadian System of Soil Classification) and were underlain by bedrock or buried talus.

### Experimental manipulation

To examine abiotic and biotic influences of shrub canopies, we established and maintained six replicate plots over 3 years for each of the following four treatments: (1) intact tall shrubs (hereafter referred to as “shrub”), (2) adjacent tall shrub-free tundra (hereafter referred to as “open tundra”), (3) artificial canopies, and (4) canopy removals (Fig. 1). In September 2007, we constructed artificial canopy plots and canopy removal plots of 6 m in diameter, similar in size to shrub patches in the study area. The mean shrub height for all plots was 65 ± 4 cm in 2008 and 76 ± 4 cm in 2009 for the natural shrub treatment, and 47 ± 4 cm in 2008 and 60 ± 7 cm in 2009 for the artificial canopy treatment. As artificial canopies lacked foliage, these plots were covered by 60% knitted green shade cloth to mimic natural canopy shading for approximately 2 months each year. The shade cloth treatment was implemented from 1 July 2008 to 7 September 2008 and 1 July 2009 to 5 September 2009.

### Conditions prior to manipulation

To examine differences in plots prior to experimental manipulation, we measured aboveground biomass, soil properties and carbon and nitrogen content in plants and soils. We harvested aboveground vegetation in August 2007 to quantify the biomass of shrub and understory species. Two 50 × 50 cm subplots were harvested 1 m up- and downslope and 1 m adjacent to the center of each the 24 treatment plots. Biomass samples were sorted into the following plant functional group categories: tall shrub species (the *Salix* and *Betula* species that typically have a growth form taller than 10 cm), prostrate shrub species (the shrub species that typically have a growth form shorter than 10 cm), graminoids (live and dead), *Dryas* (live and dead), *Cassiope*, green moss and liverworts, lichens, fungus, forbs, brown moss, and decomposed litter. All biomass was dried at 65°C and then weighed.

On 21 September 2007, we dug and described soil pits and measured the depth of each soil layer according to the Canadian Soil Classification System in the same plots harvested for biomass samples. At the same time, we harvested 5 × 5 × 5 cm cubes of the top 5 cm of the soil surface, immediately below the moss layer in the center of each of the biomass harvest plots. These samples were collected, transported to the laboratory, and stored frozen. The soil samples were divided into subsamples. One set of the subsamples (2 × 5 × 5 cm cubes) were dried at 65°C, weighed for the calculation of bulk density. The other subsamples (3 × 5 × 5 cm cubes) were used for laboratory CO_2_ incubations to measure rates of soil CO_2_ respiration (following methods described in Ruess et al. 1989). We ground soil samples, subsamples of biomass from the dominant plant functional groups, and litter from the decomposition experiment. Samples were homogenized by hand and ground with a ball mill or coffee grinder. We analyzed 2–3 mg of each homogenized soil, plant, or litter sample for total carbon and nitrogen analysis using a Control Equipment Corporation Model 440 Elemental Analyzer (Chelmsford, MA).

### Abiotic factors: soil temperatures and snow depth

To measure soil temperatures, we installed Hobo Micro Station 12-bit temperature sensors (±0.1°C, HOBO, Onset Computer Corp., MA) at 2 and 5 cm below the soil surface in the center of each plot. To measure snow depth, we attached iButton Thermochron temperature loggers (±1°C, Model DS1921G, Dallas Semiconductor Corporation, Dallas, TX) to stakes at 2, 5, 25, 50, and 100 cm above the soil surface in the artificial canopy, canopy removal, control shrub, and control open tundra plots.

Snow depth was determined by comparing the daily mean temperature difference among iButtons at each height on the snow stake and air temperature (Danby and Hik 2007a). Wooden stakes were used for snow measurements during the 2007–2008 winter; however, some stakes broke. Therefore, during the winter of 2008–2009, we switched to metal stakes with each iButton sensor insulated from the metal using 1 cm-thick closed-pore sealing foam. Snow stakes were installed 1.5 m up- and downslope of the soil temperature sensors at the center of each of the treatment plots (Fig. 1). Hobo and iButton temperature loggers were also installed 1.5 m above the soil surface in a radiation shield in the center of the experimental site to measure air temperature (Fig. 1). Hobo Microstation temperature measurements were logged every 5 min, and iButton temperature measurements were logged every 6 h. We calculated thawing and freezing degree days (FDD) from temperature data. We defined thawing degree days (TDD) as the sum of the average daily degrees above freezing, and FDD as the sum of the average daily degrees below freezing during the calendar year.

### Biotic factors: decomposition, nitrogen bioavailability, soil respiration, and soil moisture

We used litter bags to measure rates of decomposition among treatment plots. We stapled 10 × 10 cm bags made out of 1 × 1 mm mesh divided into two pouches. In each side of the litter bags we inserted 0.5 g of cellulose filter paper (75 mm Whatman qualitative) or homogenized and air dried *Betula glandulosa* litter from a common site adjacent to the experimental plots. Litter bag contents were weighed to a precision of 0.01 g before installation. Litter bags were incubated for 1 year from 21 September 2007 to 26 September 2008. We placed paired litter bags on the ground surface and horizontally in the soil at 5 cm depth. Litter bags were installed 1 m up- and downslope of the center of the shrub and open tundra treatments. After removal, paper and litter samples from the litter bags were dried at 65°C and weighed to an precision of 0.01 g. Litter samples were then ground for carbon and nitrogen analysis using a mortar and pestle.

To measure ammonium and nitrate bioavailability, we installed anion and cation exchange resin probes (Plant Root Simulator™ ion exchange probes, Western Ag Innovation Inc., Saskatoon, Saskatchewan, Canada). Nitrogen adsorbtion was measured as NO_3_-N and NH_4_-N adsorbtion using ion exchange probes that were charged with 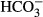 and H^+^, respectively. The probes were inserted vertically into the top 10 cm of the soil surface of each treatment plot and incubated for 2 months from 1 July to 20 August in 2007 and 1 July to 31 August in 2008. When removed, probes were cleaned with water, packaged in individual plastic bags, and shipped on ice to Western Ag Innovations for laboratory analysis.

We conducted soil CO_2_ efflux measurements using a LI6400 infrared gas analyzer (*LI-COR Environmental,* Lincoln, NE) throughout the growing season during the 3 years of the experiment. Efflux measurements were made using an LI-6400-09 Soil CO_2_ Flux Chamber placed on top of three replicate polyvinyl chloride collars installed permanently at each treatment plot into the top 3 cm of the soil. We conducted soil moisture measurements in the top 10 cm of the soil profile using a HydroSense® system (Campbell Scientific, Hyde Park, NSW, Australia). Both soil CO_2_ efflux and moisture measurements were conducted at the same time at intervals of ∼2–3 weeks throughout the growing season.

### Statistical analyses

Statistical analyses were conducted using the software R (version 2.15.2, R Development Core Team, Vienna). To test for differences among shrub and open tundra plots prior to experimental manipulation, we used multivariate analysis of variance (MANOVA). Kruskal–Wallis rank sum tests were used for biomass data that were not normally distributed and skewed by zero values.

To test whether shrub canopies insulate soils by trapping snow in winter and shading soils in summer, we compared snow depth, shading, and soil temperatures among four treatments: shrub, open tundra, artificial canopies, and canopy removals. We used mixed models (library nlme) for variables measured over multiple years and ANOVAs for variables measured once prior to manipulation. We used treatment as a fixed effect and year as a random effect in models for mean July and January temperature data at 2 and 5 cm depth, the freezing and TDD and snow depth data with Tukey post hoc tests to compare between treatments. Because snow data were not continuous, we rank transformed the snow depth on the winter day with the maximum difference in soil temperatures (8 February 2008, 7 January 2009, 2 January 2010).

To determine whether shrub canopy cover explains variation in nutrient parameters, we used ANOVAs to test for differences in total nitrogen, nitrate or ammonia absorption and litter bag decomposition among treatments. We used mixed models with Tukey post hoc tests and day of year nested within year as random effects to test for differences in CO_2_ fluxes and soil moisture that were measured on multiple occasions across each growing seasons and among years.

To explore whether abiotic or biotic factors associated with shrub canopy cover best explain variation in measured parameters, we used stepwise multiple linear regression. Explanatory variables used in temperature models included distance to shrub, mean July soil moisture, moss biomass, and organic layer depth. Variables used in nutrient models included bulk density, organic matter depth,% soil C, % soil N, soil moisture, total biomass, mean July temperature, and mean January temperature. We included only statistically independent explanatory variables (correlation coefficients of less than 0.5) in the initial models (variance inflation factors <2). Final models include all variables deemed to be significant through forward and backward stepwise model selection by Akaike information criterion. The variables soil depth, moss biomass, soil CO_2_ respiration, nitrate and ammonia adsorbtion, carbon and nitrogen content were log transformed to satisfy the assumptions of linear models.

## Results

### Conditions prior to manipulation

Shrub plots had approximately two times more live biomass, nitrogen, and carbon in the live plant biomass relative to open tundra plots (Table A1, Fig. 5). We observed statistically significant differences in mean July soil temperatures, mean soil moisture, total biomass, and biomass carbon and nitrogen (MANOVA, Pillai = 0.75, *F* = 10.79, *P* < 0.01); however, we observed no significant differences in soil depth, bulk density, organic layer depth, moss biomass, total understory biomass, total nitrogen, nitrate, ammonia adsorption, carbon respired from soil samples or mean CO_2_ flux between shrub and open tundra plots at the establishment of the experiment (MANOVA, Pillai = 0.53, *F* = 1.45, *P* = 0.26; Table A1, Fig. 5 and 7). Soil temperatures in shrub plots were on average 1.6°C cooler than in open tundra plots in July 2007 prior to the experimental manipulation. During this time, there was no significant difference in mean July soil temperatures among those shrub and open tundra plots that were retained as controls and those that underwent the subsequent experimental manipulations (ANOVA, *F*_1,22_ = 0.01, *P* = 0.90, Tukey post hoc test comparisons, *P*_*shrub control – manip. shrub*_ = 0.99, *P*_*tundra control – manip. tundra*_ = 0.99).

### Do shrub canopies insulate soils by trapping snow winter and shading soils in summer?

**Figure 2:**
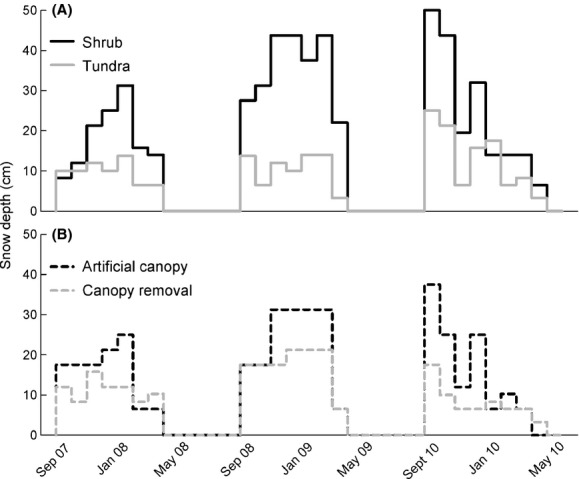
The median of snow depth at (A) shrub and open tundra plots and (B) manipulated treatments for the day with the maximum difference in soil temperatures during the 2007–2008 and 2008–2009 winters (8 February 2008, 7 January 2009, 2 January 2010; *n* = two stakes for each of six replicate plots per treatment).

**Figure 3:**
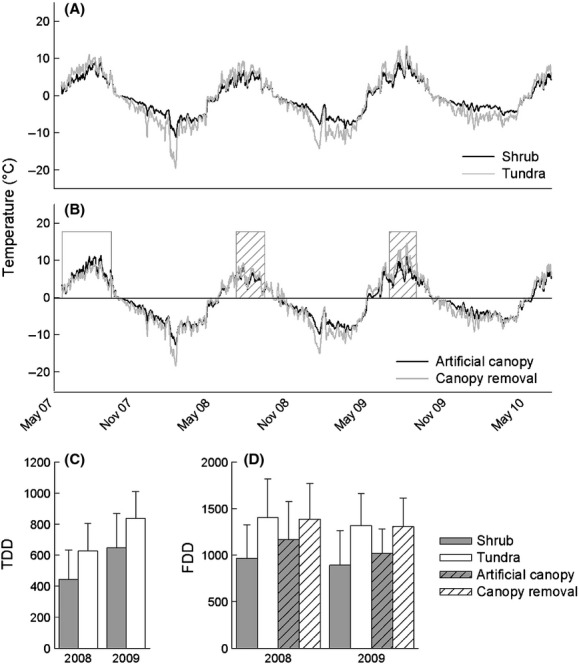
Soil temperature profiles among (A) shrub and open tundra plots, (B) manipulated treatments, (C) mean ±95% confidence interval of thawing degree days (TDD), and (D) freezing degree days (FDD) at 2 cm depth (*n* = six plots per treatment). In plot B, the open box indicates the period prior to the manipulation, where the “canopy removal” line is the mean temperature under intact shrub canopies and the “artificial canopy” line is the mean temperature in unmanipulated open tundra plots and the hatched boxes show the period when shade cloth covered the artificial canopies.

**Figure 4:**
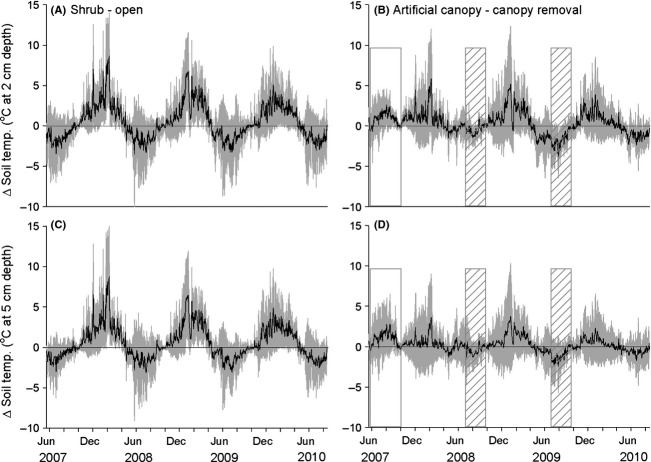
Differences in soil temperatures between the treatments (A and C for shrub minus open tundra treatments and B and D for artificial canopy minus canopy removal treatments, *n* = six plots per treatment). Black lines indicate the mean daily temperatures and gray lines the 95% confidence intervals (a and c at 2 cm depth and c and d at 5 cm depth). In plot B and D, the open box indicates the period prior to the manipulation, where the “canopy removal” line is the mean temperature under intact shrub canopies and the “artificial canopy” line is the mean temperature in unmanipulated open tundra plots and the hatched boxes show the period when shade cloth covered the artificial canopies.

Natural and artificial canopies trapped more snow than open tundra and canopy removal plots (Table 1 and 2, Fig. 2) and mean January soil temperatures were warmer in shrub versus open tundra plots, and artificial canopy plots versus canopy removal plots at 2 cm depth (Table 1 and 3, Figs. 3 and 4). Differences among the manipulated treatments were not significant at 5 cm depth (Table 1 and Figs. 3 and 4). Mean July soil temperatures were cooler in shrub compared with open tundra plots, and artificial canopy compared with canopy removal plots when the shade cloth treatment was in effect (Table 1 and 2, Figs. 3 and 4). Open tundra plots had both deeper thaw depths and greater FDD than shrub plots, though there was no significant difference among the manipulated treatments (Table 1 and 2, Fig. 3). A plot-level analysis of shrub cover, soil depths, and moss biomass indicated that the presence and proximity of the shrub canopy was a major explanatory variable describing January soil temperatures (Table 1). Greater moss biomass was associated with cooler July soil temperatures (Table 1), though moss biomass did not significantly differ among canopy, open tundra, canopy removal, or artificial canopy treatments (Table A1).

**Table 1:** Soil temperature and snow depth differences among treatments

Soil Temperature Models														
			Shrub – Tundra		Art. canopy – Can. Removal		Shrub – Art. Canopy		Art. canopy – Tundra		Shrub – Can. Removal		Can. removal – Tundra	
								
Variable	Depth	Number of observations	Est.	*P-*value	Est.	*P-*value	Est.	*P-*value	Est.	*P-*value	Est.	*P-*value	Est	*P-*value
July	2 cm	48	−2.0	<0.01**	−1.5	0.03*	−0.6	0.69	−1.4	0.05	−2.1	<0.01**	0.1	1.00
	5 cm	48	−1.9	<0.01**	−1.1	0.2	−0.8	0.46	−1.1	0.17	−1.8	<0.01**	0.0	1.00
Jan	2 cm	72	3.7	<0.01**	2.0	0.01*	1.4	0.18	2.3	<0.01**	3.4	<0.01**	0.3	0.97
	5 cm	72	3.6	<0.01**	1.3	0.19	1.4	0.14	2.2	0.01*	2.8	<0.01**	0.9	0.57
TDD	2 cm	24	−187	0.01*	Not calculated for manipulated treatments									
	5 cm	24	−160	0.03*										
FDD	2 cm	48	−430	<0.01**	−254	0.11	−165	0.47	−266	0.09	−419	<0.01**	−12	1.00
	5 cm	48	−431	<0.01**	−146	0.5	−193	0.25	−238	0.1	−339	<0.01**	−92	0.81
Snow		72	∼22	<0.01**	∼10	0.39	∼7	0.21	∼15	<0.01**	∼17	<0.01**	∼5	0.35

Art. = Artificial; Can. = Canopy.

Comparisons of soil temperature, thaw degree days (TDD) and freezing degree days (FDD) and snow depth between treatments (mixed models with Tukey post hoc tests). Snow depth comparisons are for the winter day with the maximum difference in soil temperatures in each year (8 February 2008, 7 January 2009, 2 January 2010).

*0.01–0.05, **<0.01.

**Table 2:** Factors explaining variation in soil temperatures across all plots

	Year	Initial model	Final model	Slope ± SE	df	*F-*Value	*P-*value	*R*^*2*^
Mean July	2008	Distance + Moisture	Moss**	−0.7 ± 0.2	1,22	12.1	<0.01	0.33
	2009	+ Moss + Organic	Distance + Moss*	0.1 ± 0.1 −0.6 ± 0.3	2,21	3.5	0.05	0.18
Mean Jan.	2008		Distance*	−0.3 ± 0.1	1,22	7.9	0.01	0.23
	2009		Distance** + Moisture	−0.3 ± 0.1 0.9 ± 0.7	2,21	7.2	<0.01	0.35
	2010		Distance*	−0.2 ± 0.1	1,22	7.0	0.02	0.21

Stepwise linear regressions describing variation in mean July and January soil temperatures at 2 cm depth used the variables distance to shrub, mean July soil moisture, moss biomass, and organic layer depth. The minimum distance to shrub canopy and snow depth could not both be considered in these models as they were highly correlated; however, in individual regressions, the minimum distance to shrub canopy from the snow stakes was negatively correlated (linear mixed model, df = 68, *t* = −4.70, *P* < 0.01) and snow depth was positively correlated with mean January soil temperatures at 2 cm depth (linear mixed model, df = 68, *t* = 4.34, *P* < 0.01).

*0.01–0.05, **<0.01.

**Table 3:** Factors explaining variation in nutrient variables across all plots

Data set	Dependent variables	Initial model	Final model	Slope ± SE	*DF*	Adj. *R*^2^	*F-*value	*P-*value
Incubations	Day 7, Day 14, Day 25	Bulk density +%C + biomass + July temp.	ns					
N Probes	2007 Total, 2007 NO_3_, 2007 NH_4_, 2008 NO_3_, 2008 NH_4_	Organic matter +%N soil + July temp. + moisture	ns					
	2008 Total		July temp.*	0.08 ± 0.03	1,22	0.18	6.20	0.02
Litter Bags	Litter 0 cm, Paper 0 cm, Litter 5 cm	Bulk density +%N soil + biomass + January temp. + moisture	ns					
	Paper 5 cm		Bulk density* +%N soil** + Jan. temp.* + moisture	−0.17 ± 0.06 10.59 ± 2.90 1.00 ± 0.43 −0.075 ± 0.05	3,20	0.51	6.95	<0.01
CO_2_ Flux		Organic matter +%C soil + biomass + July temp. + moisture	%C soil + July temp.	0.02 ± 0.01 0.09 ± 0.05	2,21	0.20	3.80	0.04
Soil Moisture		Bulk density + July temp.	ns					

Stepwise multiple linear regressions describing variation in soil CO_2_ respired during incubations, nitrogen adsorbtion, decomposition of litter bag treatments, and measured CO_2_ and soil moisture for all plots using soil, biomass, temperature, and moisture explanatory variables.

*0.01–0.05, **<0.01.

### Does shrub canopy cover explain variation in nutrient parameters?

We found little evidence that shrub canopy cover explained variation in the nutrient parameters measured in this study. In the litter bag incubations, we observed lower mass loss of the filter paper substrate at the soil surface in all plots (Fig. 6), and higher decomposition of the paper substrate in the shrub plots at 5 cm depth when compared with the other treatments (Fig. 6, ANOVA, *F*_3,20_ = 4.02, *P* = 0.02). The percent carbon and nitrogen in the litter substrate after decomposition was the same with the exception of percent carbon in the litter bags deployed on the soil surface, which was lower in shrub versus artificial canopy and canopy removal plots (Fig. 6, ANOVA, *F*_3,20_ = 4.91, *P* = 0.01). We found no significant difference in nitrate or ammonia adsorption (Fig. 7) and only observed significantly higher total nitrogen in the canopy removal plots (ANOVA, *F*_3,20_ = 4.64, *P* = 0.01). And finally, there was no significant difference in field and laboratory measurements of respired CO_2_ among treatments (Fig. 8, mixed model, all Tukey post hoc test comparisons = ns).

### Do abiotic or biotic factors associated with shrub canopy cover best explain variation in nutrient dynamics?

We found weak relationships among soil temperatures and variation in the nutrient parameters measured in this study. July soil temperature at 2 cm depth explained 19% of the variation in total nitrogen adsorbtion across all plots (Table 3). Field measurements of CO_2_ soil efflux were weakly associated with the variables soil percent carbon, and mean July soil temperature at 2 cm depth (Table 3). Only eight percent of the variation in the field measurements of CO_2_ soil efflux was explained by soil temperatures and none of the variation was explained by soil moisture measurements taken at the time of the flux measurements (Stepwise Linear Regression, *F*_1,289_ = 26.5, *R*^*2*^ = 0.08, *P* < 0.01). The only significant model for the decomposition data showed that soil bulk density and soil percent nitrogen explained 42% of the variation in decomposition among plots for the paper substrate at 5 cm depth (Table 3).

## Discussion

Our results suggest that although shrub canopy cover influenced soil temperatures, the abiotic effects of canopy cover only weakly influenced the nutrient dynamics. Our results confirm that shrubs trap snow in areas where it is redistributed by wind, and that increased snowpack insulates soils in winter, while in summer shading from shrub canopies cool soils. Under shrub canopies, the 2°C cooler temperatures during the most biologically active time of year is substantial as compared with the 4–5°C warmer temperatures observed during the coldest part of the winter. Although several observational studies document differences in nutrient cycling between tall shrub and tall shrub-free tundra plots (Myers-Smith et al. 2011a), we found weak or no influence of canopy manipulation treatments on the nutrient parameters measured in this study. Tall shrub canopies will likely alter tundra nutrient cycling over the long term due to biotic factors such as litter inputs (Buckeridge et al. 2010), course woody debris (De Deyn et al. 2008), soil biota (Chu et al. 2011), and the balance of carbon and nitrogen stores (Mack et al. 2004; Weintraub and Schimel 2005). However, without evidence of short-term abiotic influences of tall shrub canopies on tundra ecosystem functions, feedbacks to climate warming and further shrub expansion could in fact be weaker than are commonly asserted. Further experimentation using artificial shrub canopies and canopy removals is required to mechanistically understand and quantify shrub-snow-shading-nutrient feedbacks and the ecosystem consequences of future shrub expansion.

### Experimental canopy treatments

Snow fence experiments have been frequently used to examine the influence of snow cover on tundra phenology, productivity, community composition, and nutrient cycling (Wipf and Rixen 2010). However, many snow fence experiments do not simulate snow cover scenarios that are representative of snow trapping by tall shrub canopies. In a review of snow experiments, fence manipulations were found to increase snow depth on the order of 2 ± 1 m (Wipf and Rixen 2010), whereas shrub canopies in this study only increased snow depths by ∼25 cm (Fig. 2). Artificial canopies and canopy removals provide more realistic snow addition treatments; however, these manipulations also have their caveats. Our manipulation was maintained for 3 years, but if the experiment continued over a longer period, the differences in soil temperatures could increase overtime as the microclimatic influences of the canopy treatments infiltrate deeper into the soil profile. In addition, plant community composition would likely change in the experimental treatments, and the biotic influences of canopies and canopy removals could become more important over time.

Temperature differences between artificial canopies and canopy-free treatments were weaker than those for unmanipulated shrub canopies and open tundra plots. These canopies were formed with dead stems fastened to the soil surface, rather than being rooted in the soil, and by spring, some stems had fallen over. The artificial canopies were therefore lower, less dense and likely had reduced strength to trap and hold snow during winter. These factors could explain the lower snow depths and cooler winter soil temperatures observed in the artificial canopy treatment. Alternatively, the artificial canopy plots might have been located in sites that had lower snow depths due to localized topography. Likewise, although light penetration was similar between natural and artificial canopy treatments in summer, the shade cloth did not completely replicate leaves and this could explain the slightly warmer soils in the artificial canopy versus tall shrub plots.

Plant removal experiments can create disturbances that can influence the resource supplies and habitat structure for remaining organisms including for example physical, chemical, or biotic alteration of the soil (Díaz et al. 2003). In our study, the canopy removal treatment did not replicate the biotic environment of open tundra as the canopy removals exposed an understory primarily composed of litter and bare soil in many of the plots. These plots had a dark surface and therefore warmed substantially during the summer relative to the other experimental treatments. We observed greater total nitrogen adsorbtion (NO_3_-N + NH_4_-N) in the canopy removal treatments in 2008, which could be related to warmer temperatures experienced during summer in those plots (Table 1), reduced plant uptake and/or increased addition of fine root litter inputs and associated loss of mycorrhizal function caused by the canopy removal (Bardgett et al. 1998). However, high nitrogen adsorbtion was also observed in these experimental plots prior to manipulation (Fig. 7). Small mammals provide the largest point source of nitrogen in this system (I. H. Myers-Smith and D. S. Hik, unpubl. data), so the presence of small mammals or variability in soil nitrogen pools could also account for the greater nitrogen adsorbtion in the plots that underwent the canopy removal manipulation.

### Litter decomposition and negative shrub–climate feedbacks

Greater snow depths and warming winter soil temperatures could lead to enhanced decomposition (Baptist et al. 2010); however, experimental investigations of winter warming events and reduced snowpack have not always shown changes in litter decomposition (Bokhorst et al. 2010). Our data did not provide evidence that tall shrub canopies, and resulting soil insulation due to snow trapping, influence the rate of decomposition over a 1-year incubation. We did, however, observe greater decomposition of paper at 5 cm depth in shrub plots (Fig. 6). The shrub plots experienced cooler soils in summer and warmer soils in winter and had deeper snow depths; however, mean January soil temperature was only one of the four explanatory variables that best described the variation in paper decomposition. We did not observe greater paper decomposition at 5 cm depth in artificial shrub plots which also trapped snow and had warmer soil temperatures over winter. The observed greater decomposition in soils under natural shrub canopies could be an indication of greater cellulitic decomposition due to biotic factors such as a priming effect of greater fine root turnover in shrub plots or a different decomposer community (Hartley et al. 2010; Chu et al. 2011).

### Seasonal variation in nutrient dynamics

Seasonal variation could be a key element in explaining shrub–temperature–nutrient dynamics. Increased snow cover has been shown to promote higher levels of microbial nitrogen immobilization over winter, greater nitrogen fluxes in spring and potentially greater uptake by vegetation at the beginning of the growing season (Brooks et al. 2011). Our study site was not accessible in winter and early spring. Without year round measurements of nutrient parameters, we were not able to quantify how nutrient cycling rates vary seasonally, nor could we calculate annual nutrient budgets.

### Differences over time and across the landscape

The influence of tall shrub canopies on winter warming, snow duration, and summer cooling is moderated by weather conditions in a given year (Pomeroy et al. 2006) and will vary with different extents of shrub cover. Differences in snow depth among treatments were larger in the high snowfall winters of 2008–2009 and 2009–2010 (Environment Canada, Burwash Weather Station). Differences in summer cooling among shrub tundra, artificial canopies, open tundra, and canopy removal treatments were greater in 2009 (one of the warmest summers in recent years (Environment Canada, Burwash Weather Station). The influences of tall shrub canopies in regulating the soil microclimate could become more important with greater variability in snow fall and temperatures.

Multiple factors will interact to alter the effects of tall shrub canopies on understory vegetation and soil temperatures. Winter insulation is controlled by canopy height, structure, stem bending, and snow-loading capacity in addition to snowpack development, wind, and landscape topography (Sturm et al. 2001a, 2005; Liston et al. 2002; Marsh et al. 2010), and summer shading by the height and density of the canopy (Pomeroy et al. 2006; Brantley and Young 2010). We found significant differences in soil moisture between shrub and open tundra plots prior to the experimental manipulation (Fig. 8) indicating that evapotranspiration and canopy cover could be influencing water and latent heat fluxes in this ecosystem. Understanding the relative importance of the winter warming and summer cooling influences of shrub canopies, particularly in the context of other factors such as snowpack duration or soil moisture, will be critically important when modeling the influence of tall shrubs on tundra ecosystem functions such as soil carbon storage, nitrogen cycling, or permafrost degradation (Liston et al. 2002; Myers-Smith et al. 2011a).

The influence of tall shrub canopies will likely vary with shrub cover, density, and canopy height. In areas of dense shrub cover, shrub-induced summer cooling will likely dominate winter warming, as snow redistribution should be minimal (Lantz 2008). Our study indicates that where shrubs occupy about half of the ground surface, canopies insulate soil temperatures in winter and shade soil temperatures in summer. In zones of sparse tall shrub cover, both shading and snow trapping will likely be negligible. In addition, the spatial arrangement of shrub cover will influence the distribution of snow and resulting soil insulation (Lantz 2008). Furthermore, the ecological impacts of increasing shrub cover will likely vary with species, growth form, and site conditions (Myers-Smith et al. 2011a).

## Conclusion

Tall shrubs are foundational species altering tundra ecosystem functions. The snow–shrub hypothesis predicts that expansion of shrubs into tundra ecosystems will create a positive feedback through snow trapping, temperature warming, and enhanced nutrient cycling to promote further shrub growth. In our experiment, the short-term effects abiotic of canopy cover did not explain variation in soil litter decomposition, carbon fluxes, and nitrate or ammonia adsorbtion. Although shrubs trapped snow and soil temperatures were warmer in winter under both natural shrubs and artificial canopies, shrubs also shaded soils resulting in cooler summer soil temperatures. Our results suggest that abiotic influences could be less important than the biotic effects of shrub canopies on nutrient dynamics in tundra ecosystems.

## Figures and Tables

**Figure A1:**
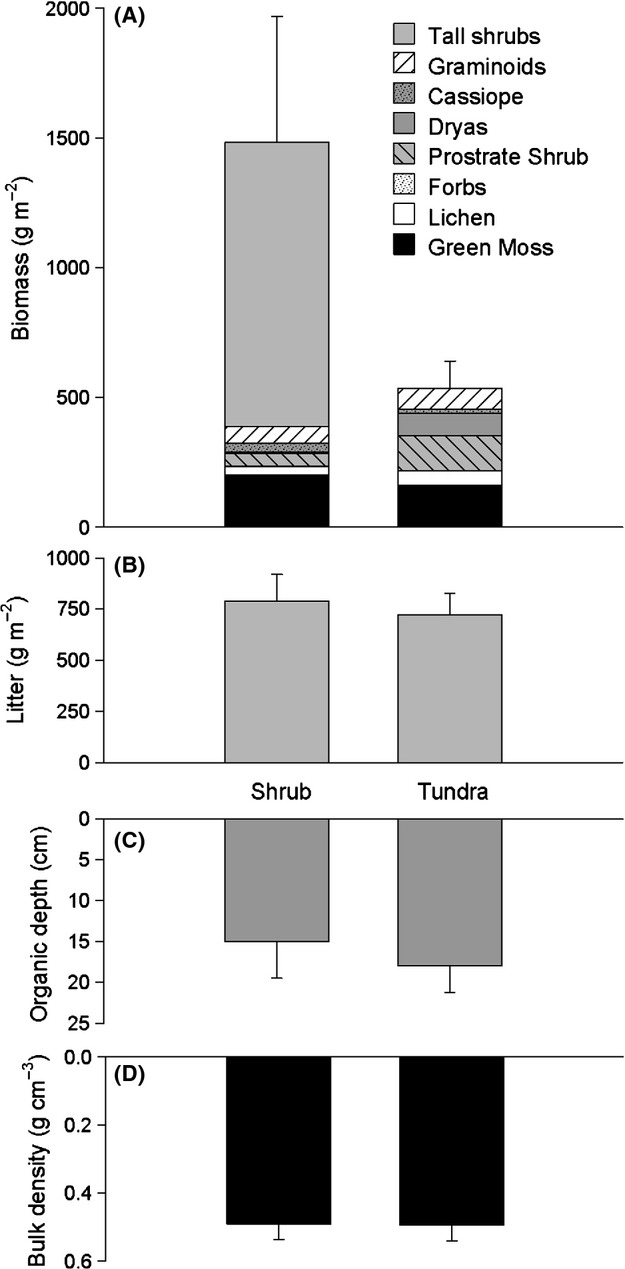
Biomass of plant functional groups (A), litter (B), soil layer depths (C) and soil bulk density (D) in shrub and open tundra plots prior to the experimental manipulation (*n* = two vegetation or soil harvests for each of 12 plots per vegetation type). Error bars indicate 95% confidence intervals.

**Figure A2:**
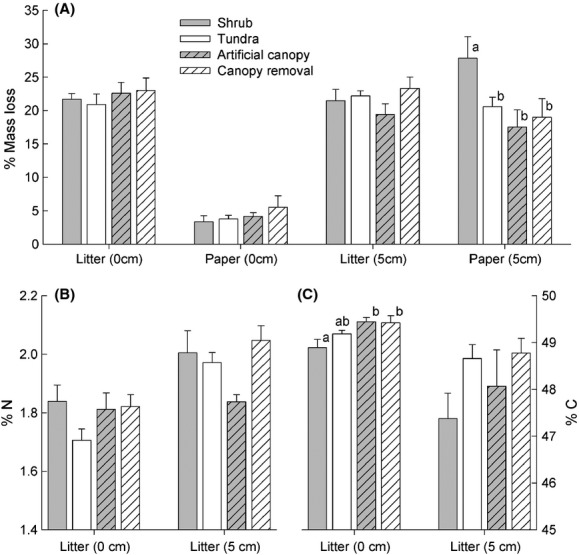
Percent mass loss among the litter and paper substrates over 1 year from 21 September 2007 to 26 September 2008, on the surface and at 5 cm depth in the soil profile (A), percent nitrogen in leaf litter after incubation (B), and percent carbon in leaf litter after incubation (C, *n* = two litter bags for each of six plots per treatment). Letters indicate significant differences among treatments for litter mass loss at 5 cm depth and percent carbon in incubated litter at the soil surface. Error bars indicate 95% confidence intervals.

**Figure A3:**
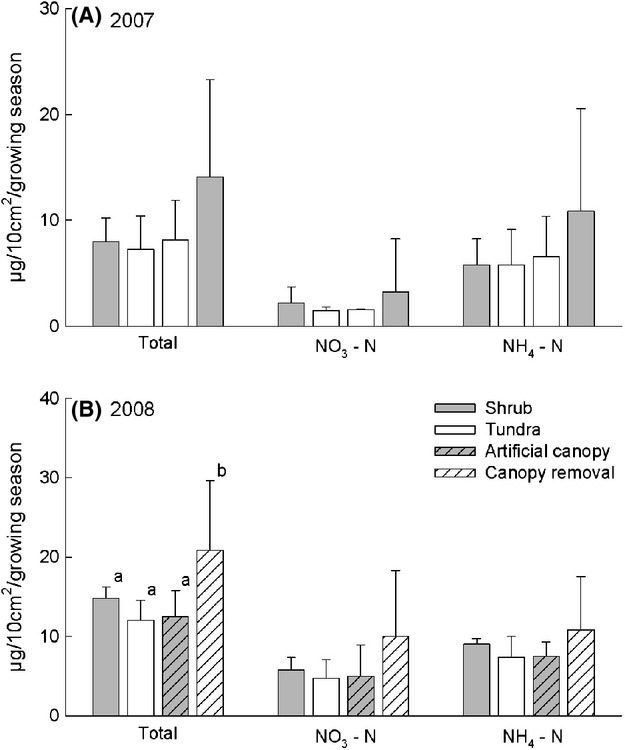
Accumulation of nitrate and ammonium on the membrane of PRS™ probes in each of the treatment plots across the growing seasons of (A) 2007 and (B) 2008 (*n* = two probes for each of six plots per treatment). In 2007, incubations were conducted prior to the establishment of the experimental treatments; therefore, plot A) shows the bars for the control shrub and tundra plots and the shrub and tundra plots that were assigned to the experimental treatments. A significant difference in total accumulation (nitrate plus ammonia) was observed among treatments in 2008 as indicated by the letters, no significant difference between treatment plots was observed in 2007. Error bars indicate 95% confidence intervals.

**Figure A4:**
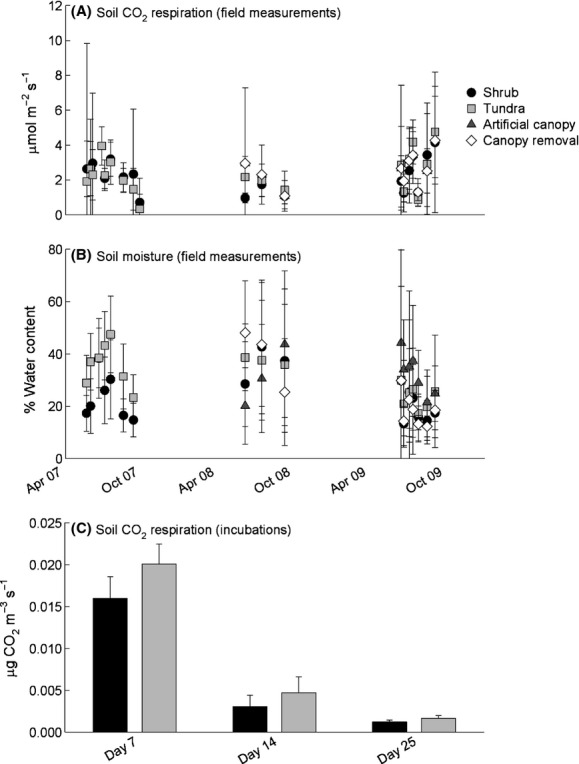
Field measurements of soil CO_2_ respiration (A) and soil moisture (B) for 2007, 2008, and 2009 for each of the experimental treatments, and laboratory measurements of soil CO_2_ respiration from 25-day incubations at 20°C (C, *n* = three measurements for each of six replicate plots per treatment). Error bars indicate 95% confidence intervals

## Appendix: Additional Methods, Tables and Figures

### Experimental manipulation

Artificial canopy and canopy removal treatments were circular in shape, and approximately 6 m in diameter if located in large shrub patches (plots 1, 3, 4, 6) or the size of the removed shrub patch (plots 2, 5). We measured the distances to surrounding shrub canopies from soil temperature and snow depth sensors at each plot. Because the natural and artificial canopy treatments were not continuous in cover, and some of the natural tundra controls, though nearly shrub free, had some small-in-stature tall shrub individuals growing in them; therefore, the distance to the nearest shrub canopy differs for all plots.

Artificial canopies were created by affixing the aboveground stems of the tall shrubs harvested from the canopy removal plots to small wooden fences running along the ground. The wooden fences were no more than 20 cm high and the fence posts were inserted no more than 20 cm into the ground at approximately 1 m intervals. The artificial shrub patches were constructed to have similar densities and canopy structure using the same stems harvested from the adjacent canopy removal treatment. We were not able to exactly duplicate natural shrub canopies and the artificial canopies had lower canopy height, slightly different stem spacing, and reduced stem flexibility. Over each growing season, we clipped new growth from the canopy removal plots and maintained the artificial canopies by adding new stems from outside of the research site and reerecting stems that had fallen over or broken during the course of the experiment.

### Shade cloth treatments

To establish whether shade cloth mimicked the shading of natural shrub canopies, we recorded light penetration through each of the natural and artificial canopies using a multisensor quantum light meter measuring photosynthetically active radiation (PAR; Spectrum Technologies, Plainfield, IL). Measurements were taken between 12:30 and 13:30 during peak radiation (1000–1400 μmol m^2^ s^−1^) on 4 July and 14 August 2009, both cloud-free days. We found no difference in light penetration between natural and artificial treatments, though the spectral properties of this light will likely differ (mean percent difference ±SE between incoming PAR and PAR at ground level for treatment plots: shrub = 89 ± 5%, artificial canopy = 87 ± 4%, tundra = 14 ± 6%, canopy removal = 8 ± 3%; ANOVA, *F*_1,10_ = 0.08, *P* = 0.78).

### Temperature data gap filling

During the course of the experiment, wires between sensors and data loggers at four plots were chewed by animals or damaged during maintenance of the shrub removal treatment. We repaired all damaged wires within 2 weeks, except for the sensor at 2 cm depth at the tundra plot 2A that could not be fixed and stopped logging measurements on 27 July 2008. To calculate monthly means and annual projections, we interpolated missing data by projecting temperatures from regressions between soil temperature data measured at the same location but a different depth or the closest plot with the same treatment and same depth. Regression relationships used to fill the data gaps had *R*^*2*^ of greater than 0.80.

### Snow depth calculations

For the calculation of snow depth, we assumed that a temperature difference of greater than 3°C indicated that the iButton sensor was located in the snowpack, if the sensor was reading a temperature below freezing and if all sensors located below also met the same criteria. Snow depths were first measured as intervals (less than 2, 2–5, 5–25, 25–50, 50–100, greater than 100 cm) with the median temperature of the two replicate stakes used for further analysis. During the course of each winter, some iButtons failed or fell off their stakes (33 iButtons in 2007–2008 and 10 in 2008–2009 and 36 in 2009–2010). In these instances, we used the data from the iButton placed at the same height on the replicate stake, or if these data were also missing, increased the snow depth interval to account for lack of measurement at the height of the missing sensor. As the snow depth data are not continuous, we present central tendencies using medians.

### Soil incubations

For soil incubations, we used 5 × 5 × 3 cm frozen blocks collected from surface soils from each of our treatment plots. Samples were incubated in an environmentally controlled chamber (University of Alberta Department of Biological Sciences Biotron facility) for 20 h of full light, a humidity of 50%, and a temperature of 20°C for 25 days. On 8 July 2008, we weighed and then placed the frozen blocks of soil on top of a sponge (approx. 5 × 4 × 4 cm) wetted with 30 ml of distilled water in 54 mason jars (900 mL) in the growth chamber. Six randomly chosen jars were designated control jars and contained a wetted sponge, vial of 1M NaOH, but no soil. After 7 days of incubation, CO_2_ effluxes were determined by two titrations of the 10 mL 1 mol/L NaOH that was incubated with the soil blocks. The soil titrations were repeated after 14 and 25 days on 22 July and 25 August 2008. After the final titration, the blocks of soil were dried at 65°C and ground for carbon and nitrogen analysis.

### Soil CO_2_ efflux measurements

Three repeat efflux measurements were conducted at each of the three collar locations at each treatment plot, during daylight hours between 9:00 and 21:00. The LI6400 was calibrated using soda lime CO_2_ scrub and a 397 ppm CO_2_ reference gas before each measurement campaign. We conducted measurements at intervals of approximately 2–3 weeks across the growing season on 8 days in 2007 (22 May, 31 May, 6 June, 4 July, 18 July, 18 August, 11 September, 26 September), 3 days in 2008 (6 June, 15 July, 8 September), and 7 days in 2009 (15 June, 21 June, 4 July, 13 July, 25 July, 16 August, 4 September). For some of the dates at the beginning and end of the growing season, when efflux measurements were slower, we were only able to complete a subset of the plots.

### Conditions prior to the experimental manipulations

**Table A1:** Biomass and nitrogen and carbon content among shrub and open tundra plots

	Plot type	Litter	Green Moss	Lichen	Graminoids	Prostrate shrubs	*Dryas*	Forbs	*Cassiope*	Canopy-forming Shrub	Total
Biomass g m^−2^	Shrub	790 ± 59	201 ± 58	31 ± 8*	65 ± 12	50 ± 23*	5 ± 3*	1 ± 1	33 ± 22	1095 ± 251**	2269 ± 241**
	Tundra	720 ± 48	162 ± 43	56 ± 9*	81 ± 12	134 ± 29*	86 ± 21*	∼0	16 ± 9	∼0**	1256 ± 73**
% N	Shrub	1.7 ± 0.1**	1.1 ± 0.1**	1.5 ± 0.2*	1.2 ± 0.1*	1.4 ± 0.1	1.3 ± 0.2	2.0 ± 0.4	1.1 ± 0.1	1.5 ± 0.1	
	Tundra	1.3 ± 0.1**	0.9 ± 0.0**	0.9 ± 0.1*	1.0 ± 0.0*	1.3 ± 0.1	1.2 ± 0.0	1.6 ± 0.2	1.1 ± 0.0	1	
% C	Shrub	44 ± 1	42 ± 0	40 ± 1	41 ± 1	48 ± 0	47 ± 1	44 ± 3	52 ± 1	50 ± 0	
	Tundra	43 ± 0	42 ± 0	41 ± 0	42 ± 0	49 ± 0	47 ± 0	44 ± 0	52 ± 0	45	
g N m^−2^	Shrub	13.5 ± 1.2**	2.1 ± 0.5	0.4 ± 0.1	0.8 ± 0.2	0.6 ± 0.3*	0.1 ± 0.0*	∼0	0.3 ± 0.2	15.9 ± 3.5	33.7 ± 3.9**
	Tundra	9.5 ± 0.6**	1.4 ± 0.4	0.5 ± 0.1	0.8 ± 0.1	1.6 ± 0.3*	1.0 ± 0.2*	∼0	0.2 ± 0.1	∼0	15.0 ± 0.9**
g C m^−2^	Shrub	348 ± 30	85 ± 25	13 ± 4	27 ± 5	25 ± 12**	2 ± 1**	∼0	17 ± 12	556 ± 131**	1074 ± 131**
	Tundra	310 ± 20	69 ± 18	23 ± 4	34 ± 5	67 ± 15**	41 ± 10**	∼0	9 ± 5	∼0**	552 ± 32**

Biomass, %C, %N and total carbon and total nitrogen in biomass for shrub and open tundra plots at peak biomass in August of 2007 prior to the establishment of canopy removal and artificial canopy treatments. Asterisks indicate a significant difference between shrub and open tundra plots (Kruskal–Wallis rank sum tests).

*0.01–0.05, **<0.01.
